# Risperidone/Randomly Methylated β-Cyclodextrin Inclusion Complex—Compatibility Study with Pharmaceutical Excipients

**DOI:** 10.3390/molecules26061690

**Published:** 2021-03-17

**Authors:** Laura Sbârcea, Ionuț-Mihai Tănase, Adriana Ledeți, Denisa Cîrcioban, Gabriela Vlase, Paul Barvinschi, Marinela Miclău, Renata-Maria Văruţ, Oana Suciu, Ionuț Ledeți

**Affiliations:** 1 Department Pharmacy I, Faculty of Pharmacy, “Victor Babeş” University of Medicine and Pharmacy, 2 Eftimie Murgu Square, 300041 Timisoara, Romania; sbarcea.laura@umft.ro (L.S.); circioban.denisa@umft.ro (D.C.); ionut.ledeti@umft.ro (I.L.); 2 Advanced Instrumental Screening Center, Faculty of Pharmacy, “Victor Babes” University of Medicine and Pharmacy, Romania, 2 Eftimie Murgu Square, 300041 Timisoara, Romania; 3 Faculty of Industrial Chemistry and Environmental Engineering, Politehnica University of Timisoara, Vasile Parvan Street 6, 300223 Timisoara, Romania; ionut.tanase@student.upt.ro; 4 Research Centre for Thermal Analysis in Environmental Problems, West University of Timisoara, Pestalozzi Street 16, 300115 Timisoara, Romania; gabriela.vlase@e-uvt.ro; 5 Faculty of Physics, West University of Timisoara, 4 Vasile Parvan Blvd, 300223 Timisoara, Romania; pc_barvi@yahoo.fr; 6 National Institute for Research and Development in Electrochemistry and Condensed Matter, Dr. A. Păunescu-Podeanu 144, 300587 Timisoara, Romania; marinela.miclau@gmail.com; 7 Department of Physical-Chemistry, University of Medicine and Pharmacy Craiova, 2–4 Petru Rares Str., 200349 Craiova, Romania; rennata_maria@yahoo.com; 8 Department of Medicine XIV, Faculty of Medicine, “Victor Babeş” University of Medicine and Pharmacy, 2 Eftimie Murgu Square, 300041 Timisoara, Romania

**Keywords:** risperidone, inclusion complex, randomly methylated β-cyclodextrin, compatibility studies, excipients, solubility, stability

## Abstract

Risperidone (RSP) is an atypical antipsychotic drug used in treating schizophrenia, behavioral, and psychological symptoms of dementia and irritability associated with autism. The drug substance is practically insoluble in water and exhibits high lipophilicity. It also presents incompatibilities with pharmaceutical excipients such as magnesium stearate, lactose, and cellulose microcrystalline. RSP encapsulation by randomly methylated β-cyclodextrin (RM-β-CD) was performed in order to enhance drug solubility and stability and improve its biopharmaceutical profile. The inclusion complex formation was evaluated using thermal methods, powder X-ray diffractometry (PXRD), universal-attenuated total reflectance Fourier transform infrared (UATR-FTIR), UV spectroscopy, and saturation solubility studies. The 1:1 stoichiometry ratio and the apparent stability constant of the inclusion complex were determined by means of the phase solubility method. The compatibility between the supramolecular adduct and pharmaceutical excipients starch, anhydrous lactose, magnesium stearate, and cellulose microcrystalline was studied employing thermoanalytical tools (TG-thermogravimetry/DTG-derivative thermogravimetry/HF-heat flow) and spectroscopic techniques (UATR-FTIR, PXRD). The compatibility study reveals that there are no interactions between the supramolecular adduct with starch, magnesium stearate, and cellulose microcrystalline, while incompatibility with anhydrous lactose is observed even under ambient conditions. The supramolecular adduct of RSP with RM-β-CD represents a valuable candidate for further research in developing new formulations with enhanced bioavailability and stability, and the results of this study allow a pertinent selection of three excipients that can be incorporated in solid dosage forms.

## 1. Introduction

**Figure 1:**
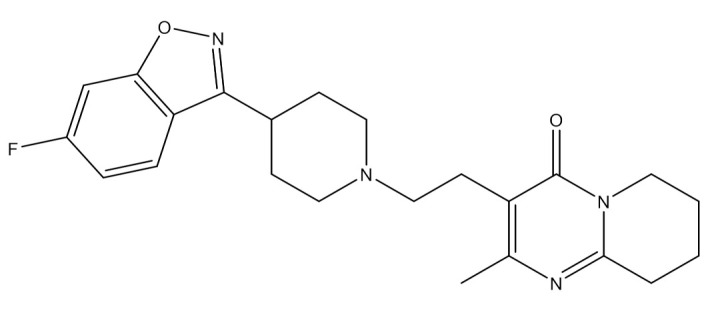
Chemical structure of risperidone.

Risperidone (RSP), 3-[2-[4-(6-fluoro-1,2-benzoxazol-3-yl)piperidin-1-yl]ethyl]-2-methyl-6,7,8,9-tetrahydropyrido[1,2-a]pyrimidin-4-one (Figure 1), is a benzoxazole derivative used in treating schizophrenia, behavioral, and psychological symptoms of dementia and irritability associated with autism [1,2]. This atypical antipsychotic drug blocks the serotonin-2 (5TH2) and dopamine-2 (D2) receptors in the brain. RSP is a member of the class of pyridopyrimdines that is practically insoluble in water and presents high lipophilicity (log *P* of 3.49), which makes it a class II candidate of the Biopharmaceutics Classification System (BCS) [1,3,4]. It exhibits the tendency of forming polymorphs [1], which could present different solubility, dissolution rates, and stability, leading to variations in the biopharmaceutical profile of its drug substance [5].

Solubility is an essential feature of drugs, being one of the most critical and important characteristics that influence their bioavailability. Among the various approaches used to enhance the solubility of BCS II class drugs, encapsulation of the active pharmaceutical substances in cyclodextrins is a valuable strategy [5].

Cyclodextrins (CDs) are cyclic glucose oligomers consisting of six, seven, or eight glucose units (α, β, and γ-cyclodextrin), linked by 1,4-α-glycosidic bonds. CDs possess a hydrophobic internal cavity that provides a microenvironment for appropriate sized molecules and a hydrophilic outer surface responsible for their water-solubility. This particular structure of CDs confers them multiple applications in the pharmaceutical field, food, cosmetics, textile, and chemistry industry based on their property of forming guest–host inclusion complexes [6,7,8,9,10]. The inclusion complexation leads to an increase in the solubility of insoluble drug substances, including the antiviral drug remdesivir [11] to improve the chemical stability, the biological activity, and the bioavailability of guest molecules, to prevent drug–excipient or drug–drug interactions, to reduce/eliminate the unpleasant taste or odors and also ocular and gastrointestinal irritation [10,12,13,14,15,16,17,18,19,20,21,22]. Therefore, the encapsulation of the drug in the CD cavity results in a remarkable improvement of physicochemical, biopharmaceutical properties, and therapeutic potential of the guest [23,24,25]. Despite its low aqueous solubility, β-cyclodextrin (β-CD) has achieved pharmaceutical relevance due to its availability, lack of toxicity, appropriate internal cavity size for a wide variety of drug substances, and economic advantages [6,21,26]. The random substitution of any β-CD hydroxyl group creates a disruption of stable hydrogen bond system around the CD rim generating an intensive enhancement of its aqueous solubility. Thus, several CD derivatives of pharmaceutical interest have been developed, among them methylated β-CD [27,28].

Several papers have reported the interaction between RSP and CDs such as β-CD, hydroxypropyl-β-CD (HP-β-CD), and methyl-β-CD, in solid state and in solution [4,29,30]. In addition, the solubility of RSP in aqueous solution of α-, β-, γ- and HP-β-CDs has been evaluated [31]. In our recent paper, we have investigated in both solution and solid state the encapsulation of RSP in two methylated CDs, heptakis(2,6-di-O-methyl)-β-cyclodextrin (DM-β-CD) and heptakis(2,3,6-tri-O-methyl)-β-cyclodextrin (TM-β-CD) [22].

The efficiency, safety, quality, and stability of pharmaceutical dosage forms, which are the result of the active pharmaceutical ingredients (API) combination with excipients, are of major importance in the drug development process. A proper formulation design involves the selection of suitable excipients; although these pharmacologically-inactive substances are considered inert molecules, during the formulation stage and/or under storage of final product, interactions may occur even in solid state, leading to a diminution of concentration of API [32,33,34]. Potential interactions between an API and excipients have to be evaluated because the incompatibilities between the components of a dosage form can affect the bioavailability, stability, potency, and safety of drug products [35,36]. According to the International Conference on Harmonisation (ICH) Q8 recommendations, a drug substance/excipient compatibility study should be evaluated as a part of pharmaceutical development [37].

Within this framework, the aim of this study was to investigate the encapsulation of RSP in randomly methylated β-CD (RM-β-CD) and to evaluate the compatibility of supramolecular adduct with selected pharmaceutical excipients. According to our knowledge, there is no study focused on RSP inclusion complex compatibility with excipients. In the present paper, the RSP/RM-β-CD inclusion complex has been characterized using solubility studies, thermal methods, spectroscopic techniques, and molecular modeling studies. Later, the interaction between the binary system and excipients, namely starch (STR), microcrystalline cellulose (CEL), magnesium stearate (MgSTE), and anhydrous lactose (LCT) has been studied by means of thermoanalytical tools (TG—thermogravimetry/DTG—derivative thermogravimetry/HF—heat flow), powder X-ray diffractometry (PXRD), and universal attenuated total reflectance Fourier transform IR spectroscopy (UATR-FTIR).

## 2. Results and Discussion

### 2.1. Inclusion Complex Characterization

#### 2.1.1. Phase Solubility Studies

**Figure 2:**
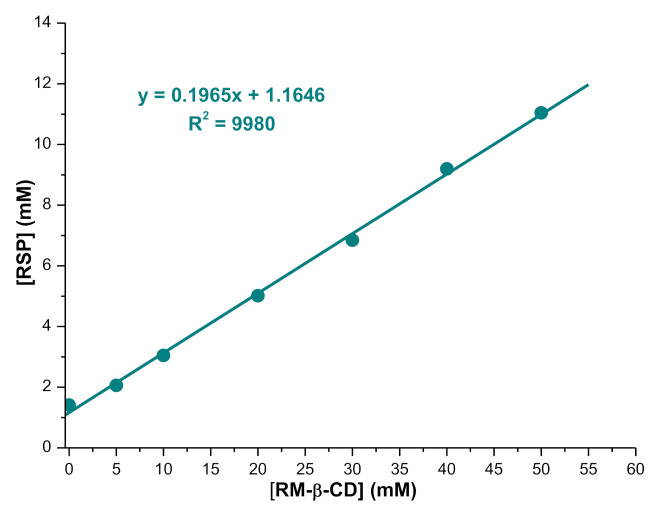
Phase solubility diagram of risperidone (RSP) in the presence of randomly methylated β-cyclodextrin (RM-β-CD) in phosphate buffer 0.1 M, pH 7.4, at 25 °C.

The stoichiometry of the RSP/RM-β-CD inclusion complex and its stability constant were investigated by means of phase solubility studies. The phase solubility diagram of RSP with RM-β-CD was obtained at 25 °C by plotting the apparent equilibrium concentration of the drug against RM-β-CD concentration. The apparent solubility of RSP increased linearly (R^2^ = 0.9980) as a function of RM-β-CD concentration in the 0–50 mM concentration range, as shown in Figure 2. The RSP solubility enhancement in phosphate buffer 0.1 M (pH 7.4) confirms the interaction between the drug substance and the CD. The linear relation between RSP concentration and RM-β-CD concentration indicates an *A_L_* type phase solubility diagram defined by Higuchi and Connors [38]; also, the slope value (0.1965) is less than unity, revealing that a soluble inclusion complex in 1:1 molar ratio was formed between the guest and host molecule in phosphate buffer 0.1 M (pH 7.4). The apparent stability constant (*K*_1:1_) of the inclusion complex calculated from the slope of the phase solubility diagram, using Equation (1) is 173.38 ± 5.54 M^−1^; this value is within the range of 100 and 5000, which is considered ideal for the formation of an inclusion complex that may improve the bioavailability profile [39,40].

#### 2.1.2. Molecular Modeling

Molecular modeling is a powerful tool employed by theoretical chemistry for quantitative predictions on guest–host interaction. The molecular docking analysis was performed using the Autodock 4.2.6 software together with the AutoDockTools [41]. The software applies a semi-empirical free energy force field and grid-based docking to assess conformations during docking process. The force field includes six pair-wise assessments (*V*) and an estimate of the conformational entropy lost upon binding (Δ*Sconf*):

(1) $$\Delta G=\left(V_{bound}^{L-L}-V_{unbound}^{L-L}\right)+(V_{bound}^{T-T}-V_{unbound}^{T-T})+(V_{bound}^{T-L}-V_{unbound}^{T-L}+\Delta S_{conf})$$

where *L* makes mention of the “ligand” and *T* refers to the “target” in a ligand–target docking calculation. Each of the pair-wise energetic terms includes evaluations for dispersion/repulsion, hydrogen bonding, electrostatics and desolvation [42]. Following the redocking analysis, the root-mean-square deviation (RMSD) values lower than 0.4 Å have been calculated, suggesting the robustness and repeatability of the docking analysis.

**Figure 3:**
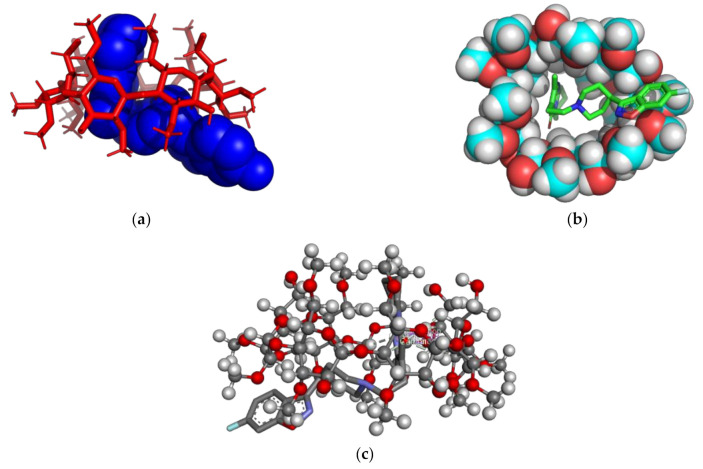
Molecular docking for 1:1 RSP/RM-β-CD inclusion complex. Figures (**a**,**b**) show the inclusion complex from the secondary face of RM-β-CD’s cavity. The RSP molecule is represented in green and blue spheres while RM-β-CD is represented in red sticks (**a**); RSP is presented in sticks colored by element, RM-β-CD is presented in spheres colored by element (**b**); Image (**c**) show contacts between RSP and RM-β-CD, RSP is colored by element, while CD is presented in balls and sticks.

The binding free energy value calculated for RSP/RM-β-CD inclusion complex (1:1) was −3.26 kcal/mol. Figure 3 presents the theoretical RSP/RM-β-CD inclusion complex, as rendered in the PyMOL [43] and Discovery Studio molecular visualization systems, simulated in a 1:1 molar ratio.

Analyzing the 3D images of the RSP/RM-β-CD (1:1) interaction, we noticed the presence of two Pi–sigma interactions, between pyrimidin-4-one cycle and the hydrogen (hydrogen methyl group) of a glucopyranose (2.28 and 2.73 Å). Two non-classical hydrogen bonds occur between the nitrogen group from the 4,5-dihydro-isoxazole heterocycle and the hydrogen from position 4 of a carbohydrate moiety. Four non-classical hydrogen bonds occur between the 1,2-oxazole heterocycle and the carbohydrate moiety hydrogen, with lengths of 2.3 Å approximately.

#### 2.1.3. Thermal Analysis

**Figure 4:**
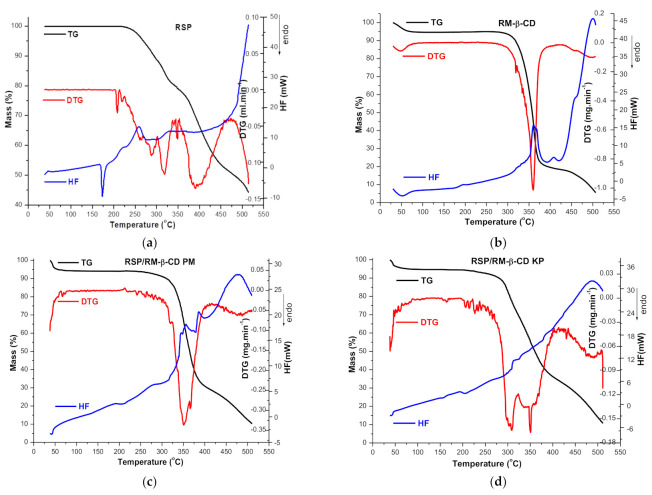
The thermal profile (TG-thermogravimetry/DTG-derivative thermogravimetry/HF-heat flow) of: RSP (**a**); RM-β-CD (**b**); RSP/RM-β-CD physical mixture (**c**); and kneaded product (**d**) in air atmosphere (100 mL/min), temperature range of 40–500 °C and heating rate of 10 °C/min.

In order to evaluate the interaction between RSP and RM-β-CD in solid state, the thermal behavior of parent substances, their physical mixture (PM), and kneaded product (KP) have been investigated using TG, DTG, and HF. The thermoanalytical curves of RSP, CD, RSP/RM-β-CD binary systems obtained by physical mixture (PM) and by kneading (KP) are shown in Figure 4a–d.

Detailed interpretation of the RSP thermal profile is presented in our previous paper [22]. The drug substance exhibits thermal stability up to 206 °C, which is a temperature that marks the onset of its decomposition; then, a continuous mass loss process is observed up to 510 °C (Δ*m* = 55.6%). As the DTG curve shows, RSP thermal decomposition takes place in three stages: the first one is noticed between 200 and 226 °C (peak at 209 °C); the second is in the temperature range of 226–350 °C (main peak at 319 °C); and the last one is between 350 and 474 °C (peak at 391 °C) [22]. RSP melting is revealed as an endothermic peak at 173 °C in the HF curve (Figure 4a) [30,44]. In addition, a small exothermic peak is noticed at 259 °C that corresponds to the first process of RSP thermal degradation; above 474 °C, the degradation occurs rapidly confirmed by the exothermic effect on HF and the rapid mass loss on the TG curve [22].

The thermoanalytical curves of RM-β-CD reveal a small mass loss (Δ*m* = 5.3%) between 40 and 85 °C and an endothermic effect (peak at 52.0 °C) which relates to the crystallization water loss (Figure 4b). A stability stage of CD is observed in the temperature range of 85–270 °C, but above 270 °C, the mass loss continues, and the degradation process takes place as the exothermic event (peak at 361.0 °C) of the HF curve indicates [19].

The thermal curves of RSP/RM-β-CD binary systems present significant differences as compared to those of the pure substances. Both the endothermic melting peak of RSP and the RSP exothermic effect are no more present neither in the HF curve of PM nor in that of KP. In addition, the endothermic events at 361.0 °C and 500 °C from the HF curve of RM-β-CD are displaced at lower temperature in HF curves of PM (357 °C and 477 °C, Figure 4c) and KP (the first event disappeared, second at 487 °C, Figure 4d). On the other hand, the TG curves of binary systems reveal a decrease in thermal stability of RSP (Δ*m* = 55.6%) in temperature range of 38–510 °C in both PM (Δ*m* = 89.7%) and KP (Δ*m* = 89.2%); this phenomenon may be a consequence of the crystallinity reduction of drug substance as a result of interaction with RM-β-CD [45]. Furthermore, the decomposition pathway of RSP in the PM and KP differs from that of pure drug substances as the DTG curves show; two distinct regions can be noticed in the DTG of KP, while the DTG of PM shows only one stage.

Thermal methods are valuable tools frequently used to investigate the interaction between CD and drug substances and to prove the encapsulation of guest molecule in the host cavity [46,47,48]. The guest–host interaction is characterized by changes in the thermal profiles of guest substances in the inclusion complex. The melting point of the guest molecule, which is embedded in the CD cavity, generally shifts to a different temperature and decreases its intensity or disappears [14,21,49]. The above-mentioned changes in the RSP thermal profile in the RSP/RM-β-CD binary products provide evidence for an interaction between the drug substance and CD as a result of inclusion complex formation.

#### 2.1.4. Powder X-ray Diffractometry

**Figure 5:**
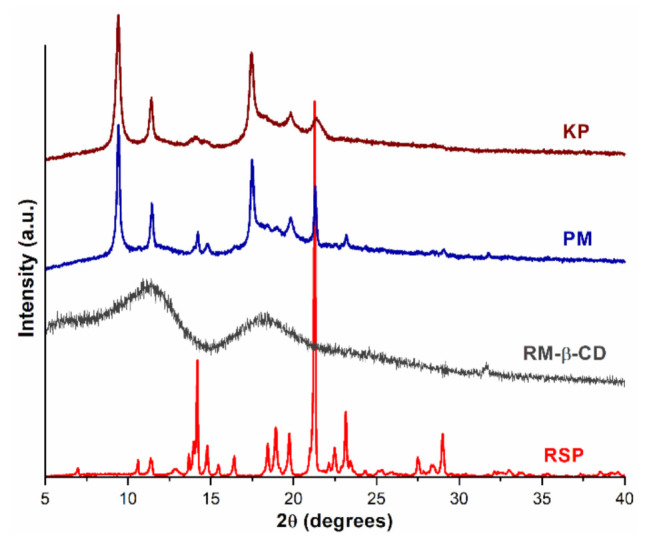
Powder X-ray diffractometry (PXRD) pattern of RSP, RM-β-CD, and RSP/RM-β-CD binary products physical mixture (PM) and kneaded product (KP).

The diffraction profiles of RSP, RM-β-CD, and their RSP/RM-β-CD PM and KP are depicted in Figure 5.

The RSP crystalline nature is emphasized by the two crystalline reflections of high intensity at 14.19 and 21.27 2*θ* in addition to other characteristic reflections at 14.79; 16.42; 18.44; 18.93; 19.74; 23.15; and 29.00 2*θ* [22]. The diffraction pattern of RM-β-CD reveals two broad peaks and many undefined, diffused peaks of low intensities, reflecting its amorphous nature [19,50]. The PXRD pattern of both RSP/RM-β-CD PM and KP present a marked diminution of RSP characteristic diffraction peaks along with the disappearance of several RSP characteristic reflections in the diffractogram of both PM (at 16.42; 18.44; 18.93 2*θ*) and KP (at 14.79; 16.42; 18.44; 18.93; 23.15 and 29.00 2*θ*), which indicate a reduction in drug crystallinity in the binary products. Furthermore, new peaks are observed in the diffraction patterns of the RSP/RM-β-CD binary systems both PM (at 9.42; 11.43; 17.40 2*θ*) and KP (at 9.42; 11.42; 17.44 2*θ*). These data suggest that interaction occurs between the drug substance and the CD and demonstrate the inclusion complex formation in solid state, confirming the results obtained using thermal methods.

#### 2.1.5. UATR-FTIR Spectroscopy

**Figure 6:**
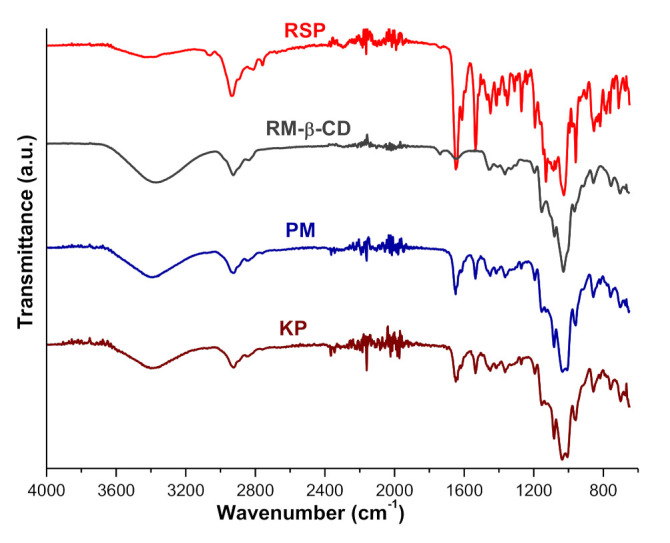
Universal-attenuated total reflectance Fourier transform infrared (UATR-FTIR) spectra of RSP, RM-β-CD, and RSP/RM-β-CD binary systems PM and KP.

Universal-attenuated total reflectance Fourier transform infrared (UATR-FTIR) spectra of RSP, RM-β-CD, and their corresponding PM and KP are presented in Figure 6.

The UATR-FTIR spectrum of RSP presents characteristic bands at 3063, 2936, 2812, 2759, 1648, 1534, 1449, 1414, 1352, 1130, 1027, 959, 854, and 816 cm^−1^ that have been assigned to the functional groups from the drug structure in our previous study [22]. RM-β-CD shows a broad absorption band in the 3600–3100 cm^−1^ region corresponding to the O-H stretching vibration from the non-methylated hydroxyl moieties and a large region below 1500 cm^−1^ which exhibits distinct peaks, which is most probably characteristic to the cyclodextrin ring [19,51].

In the spectral patterns of binary products, several differences are noticed as compared with those of the parent compounds. Thus, the band assigned to the C-N stretching vibration shifted from 1352 cm^−1^ in the RSP spectrum to 1364 cm^−1^ in both PM and KP spectra. In addition, the C=O stretching vibration (from tetrahydropyrido-pyrimidinone ring) characteristic band from 1648 cm^−1^ in the drug substance spectrum is displaced to 1649 cm^−1^ and 1642 cm^−1^ in the PM and KP spectra and is markedly reduced in intensity in both binary compounds. In addition, the spectral band attributed to aliphatic C-H stretching vibration from 2936 cm^−1^ in RSP shifted to 2929 and 2924 cm^−1^ in the spectral pattern of PM and KP, respectively. In the spectral region of 1050–1000 cm^−1^ the parent substances, RSP and RM-β-CD, exhibit bands at 1028 and 1027 cm^−1^, while in the spectral pattern of binary products, two bands can be observed, at 1009 and 1035 cm^−1^ in PM and at 1006 and 1037 cm^−1^ in KP, respectively. A marked reduction in intensity and a shift to different wavenumbers (1535 cm^−1^) in the PM and KP spectra is also noticed for the band assigned to the C=C stretching vibration of the aromatic ring from 1534 cm^−1^ in the RSP spectrum. Furthermore, the bands from 2812, 2759, and 1130 cm^−1^ in the RSP spectrum disappeared in binary KP and PM spectra.

The UATR-FTIR spectroscopy pointed out a decreasing in the intensity of RSP characteristic bands along with the shifting to different wavenumbers and the disappearance of several peaks in the spectral pattern of PM and KP. These data give evidence about the interaction between the antipsychotic drug substance and RM-β-CD.

#### 2.1.6. Solubility Profile of RSP/RM-β-CD Inclusion Complexes

**Figure 7:**
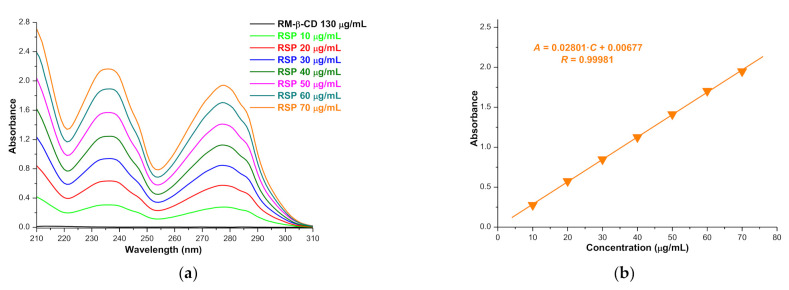
The absorption spectra of RM-β-CD and RSP in phosphate buffer 0.1 M (pH 7.4), in the spectral range of 210–310 nm, at 25 °C (**a**); RSP calibration curve at 277 nm (**b**).

The solubility of the drug substance in the inclusion complex has been evaluated using the shake-flask method [18,19,52]. The drug concentration in saturated solution was assessed using UV spectrophotometry. RM-β-CD in phosphate buffer 0.1 M (pH 7.4) does not present absorption in the spectral range of 210–310 nm (Figure 7a); an RSP calibration curve accomplished using the absorbance values from 277 nm at 25 °C (Figure 7b) was employed in order to quantify the drug in the inclusion complex.

The solubility of the included RSP as KP obtained as an average of five experimental determinations is 1392.949 ± 0.016 µg/mL. In standard controlled experiments, clear solution was obtained when 29.31 mg of RSP/RM-β-CD KP were dissolved in 5 mL 0.1 M phosphate buffer (pH 7.4) at room temperature.

The results of the saturation solubility studies reveal RM-β-CD’s ability to increase RSP solubility in phosphate buffer 0.1 M (pH 7.4) by 2.58-fold as compared with free RSP (540.007 ± 0.003 µg/mL).

### 2.2. Compatibility Studies of RSP/RM-β-CD Inclusion Complex with Excipients

The compatibility of RSP with several pharmaceutical excipients, namely microcrystalline cellulose, anhydrous lactose, starch, sodium lauryl sulfate, and magnesium stearate has been assessed and drug–excipient incompatibility has been reported in the presence of magnesium stearate, lactose, and microcrystalline cellulose [44]. In order to evaluate the ability of RM-β-CD to prevent the incompatibilities between drug substance and the mentioned excipients, compatibility studies of inclusion complex and excipients were conducted using thermal and spectroscopic techniques.

#### 2.2.1. Thermoanalytical Studies

**Figure 8:**
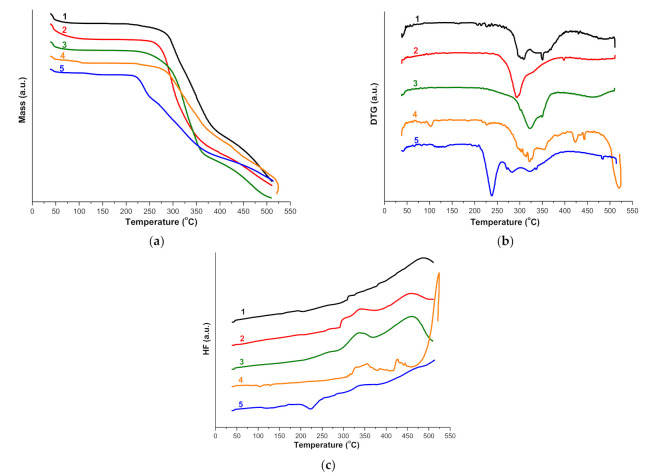
TG (**a**), DTG (**b**), and HF (**c**) curves of RSP/RM-β-CD inclusion complex (1) and its mixtures with excipients, as follows: RSP/RM-β-CD + STR (2); RSP/RM-β-CD + CEL (3); RSP/RM-β-CD + MgSTE (4); RSP/RM-β-CD + LCT (5).

The thermoanalytical TG/DTG/HF curves of the RSP/RM-β-CD inclusion complex and its mixture with pharmaceutical excipients, recorded in dynamic air atmosphere and heating rate of 10 °C·min^−1^ are presented in Figure 8a–c.

The TG/DTG curves of RSP/RM-β-CD inclusion complex suggests its thermal degradation in the following temperature ranges: 35–86 °C (Δ*m* = 5.2%, dehydration process), 197–338 °C (Δ*m* = 32%, DTG_peak_ at 309 °C), 338–414 °C (Δ*m* = 28.8%, DTG_peak_ at 351 °C) and 414–510 °C (Δ*m* = 13.2%, DTG_peak_ at 490 °C). The HF curve of inclusion complex reveals two exothermic events, a small one at 313 °C and another one at 487 °C, corresponding to the decomposition of the complex.

Regarding the inclusion complex–excipients physical mixtures, in all situations, thermoanalytical TG/DTG curves show a mass loss process at temperatures lower than 110 °C due to the release of water from complex and/or excipient. For the mixtures with CEL and LCT, the dehydration occurs at temperatures below 100 °C, as follows: RSP/RM-β-CD + CEL has water loss up to 92 °C, with Δ*m* = 4.47% and RSP/RM-β-CD + LCT has water loss up to 68 °C, with Δ*m* = 1.71%. In the case of mixtures with STR and MgSTE, the dehydration takes place up to higher temperatures, as follows: RSP/RM-β-CD + STR reaches constant mass at 124 °C, after a mass loss Δ*m* = 7.98%, while for RSP/RM-β-CD + MgSTE, the dehydration is complete at 106 °C, with a corresponding Δ*m* = 4.31% (DTG process in 94–107 °C temperature range, DTG_max_ at 103 °C).

The stability profile of mixtures with excipients in an anhydrous state is good, as revealed by the thermoanalytical curves. Accordingly to this, the RSP/RM-β-CD + STR mixture shows no thermal events in the 124–214 °C, while with the increase of thermal stress, the decomposition begins. TG/DTG curves reveal a continuous mass loss process in the temperature range 214–510 °C (DTG_max_ at 292 °C), with a corresponding Δ*m* = 77.78%. The HF curve does not reveal significant events up to 289 °C, when some exothermal processes are observed, due to the oxidative thermolysis of organic edifice (HF_max_ at 301 °C, 338 °C and 457 °C, respectively). In the case of the RSP/RM-β-CD + CEL mixture, thermoanalytical data suggest that the anhydrous mixture is stable in the 92–232 °C temperature range, since none of the three thermoanalytical curves reveal any process. In the temperature range 232–510 °C, a consistent mass loss is observed (Δ*m* = 91.37%), which is sustained by the DTG profile (main process between 233 and 391 °C, DTG_max_ at 322 °C, shoulder at 349 °C), secondary process between 391 and 510 °C, DTG_max_ at 459 °C). The HF curve reveals oxidative thermodegradation at temperatures over 233 °C, with HF_max_ at 264, 334, and 459 °C, respectively. The mixture RSP/RM-β-CD + MgSTE shows the most complex thermoanalytical profile, due to complexity of excipient composition, being known that pharmaceutical-purity MgSTE is a mix of different fatty acid salts that may vary in proportion, and additionally, its properties heavily depend on its moisture content [53,54]. After the dehydration, the RSP/RM-β-CD + MgSTE shows stability in the 106–218 °C temperature range, without revealing any interactions between the components of this matrix. The main mass loss process takes place in the 218–510 °C (Δ*m* = 81.64%), with several DTG peaks at 321, 354, 422, 444, and 519 °C, respectively), and it is accompanied by several HF peaks corresponding to an endothermic event—dehydration (96–111 °C, HF_peak_ at 104 °C), and with the increase of temperature with exothermic ones, in the 303–510 °C range: 327, 356, 428, and 524 °C, respectively. Last, for the RSP/RM-β-CD + LCT sample, the stability in anhydrous state is observed in the range 68–110 °C; then, a small mass loss takes place in the range 110–143 °C (Δ*m* = 1.65%), which is followed by the main mass loss process that takes place in the range 196–510 °C (Δ*m* = 66.18%). The DTG profile is more complex in this case, revealing peaks up to 210 °C at 63, 117, and 133 °C), and in the temperature range 200–510 °C at 238 (main), 272, 283, 322, and 482 °C, respectively). The HF curve reveals some endothermal events at 119 °C and 221 °C, while the thermal events associated with the thermooxidation of complex are no longer visible at temperatures over 300 °C. This behavior is a clear indication that some incompatibilities take place in this system, which are mainly due to the fact that they are facilitated by the presence of the melted excipient (LCT), which occurs in the range 203–243 °C (HF_peak_ at 221 °C). The stability profile of mixtures with excipients in an anhydrous state is good, as revealed by the thermoanalytical curves, and no interactions are revealed between the inclusion complex and three of the selected excipients (STR, CEL, and MgSTE), while for mixture with LCT, interactions are observed during the thermal treatment of the samples. For this last sample, a concrete evaluation of interactions can be realized solely by the implementation of two other investigational tools, namely UATR-FTIR and PXRD.

#### 2.2.2. UATR-FTIR Studies

UATR-FTIR spectroscopy is commonly used as screening technique for assessing the potential physicochemical interaction between an API and the excipients employed in the pharmaceutical dosage forms. Usually, UATR-FTIR spectroscopy is used as a complementary tool along thermal analysis for the evaluation of compatibility/incompatibility between API and excipient, which is kept under ambient conditions.

The disappearance of an absorption band, a reduction of the band intensity, or the appearance of new bands reveal the existence of interactions between the API and the studied excipient [34,55,56,57,58]. This method provides information about the chemical groups to avoid in the excipients in order to develop stable pharmaceutical formulations [55].

**Figure 9:**
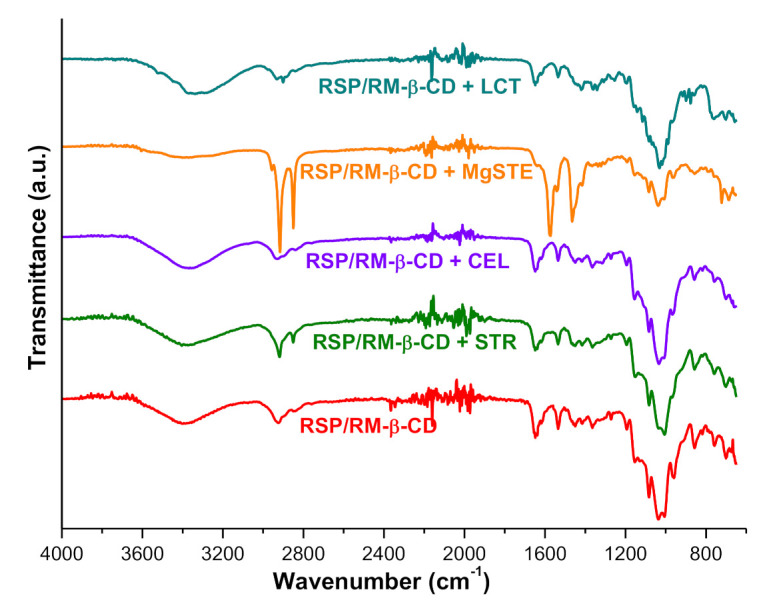
UATR-FTIR spectra of RSP/RM-β-CD inclusion complex and its physical mixtures with selected excipients.

The UATR-FTIR spectra of the inclusion complex RSP/RM-β-CD and its physical mixtures with selected excipients recorded at ambient temperature are shown in Figure 9.

The UATR-FTIR spectrum of RSP/RM-β-CD inclusion complex exhibits a broad band between 3500 and 3300 cm^−1^ (peak at 3387 cm^−1^) related to hydroxyl groups (O-H stretching vibration) and other several bands noticed in Table 1; they are presented in Section 2.1.5.

The spectral data collected in Table 1 along with the spectra depicted in Figure 1 reveal that the characteristic bands of RSP/RM-β-CD are present in the mixture of the KP with excipients either at the same wavenumber as in inclusion complex or slightly shifted to different wavenumbers, except for the case of the physical mixture of KP with LCT, where the spectral bands form 1465 and 1006 cm^−1^ have disappeared from the mixture spectrum. This situation indicates an interaction between the inclusion complex and LCT even in ambient conditions, which are results that were previously suggested by the thermoanalytical methods.

#### 2.2.3. PXRD Studies

X-ray diffractometry has been used as a complementary tool for evaluating the possible interactions between the RSP/RM-β-CD inclusion complex and the excipients, which correlate with changes in the crystallinity profile of the samples.

**Figure 10:**
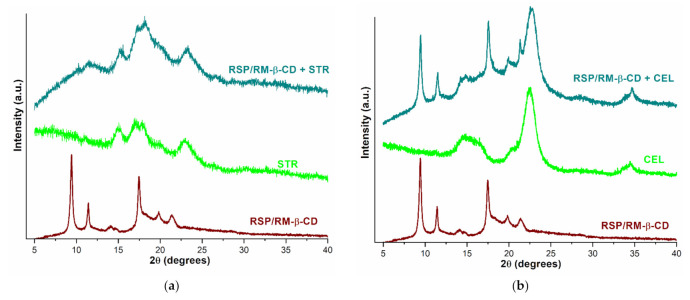

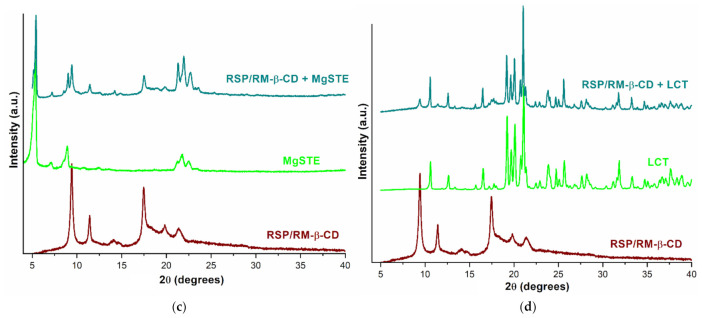
X-ray diffraction profiles of RSP/RM-β-CD inclusion complex, selected excipients, and their physical mixtures: starch (STR) (**a**); microcrystalline cellulose (CEL) (**b**); magnesium stearate (MgSTE) (**c**); lactose (LCT) (**d**).

The X-ray diffraction patterns of the RSP/RM-β-CD inclusion complex, selected excipients, and their physical mixtures are depicted in Figure 10a–d.

The diffraction profile of the RSP/RM-β-CD inclusion complex presents three characteristic reflections of higher intensity at 9.42, 11.42, and 17.48 2*θ* and others of lower intensity at 14.17, 19.87, and 21.47 2*θ*. In the diffractograms of STR and CEL, there are several main broad peaks and numerous undefined ones, with low intensities indicating the amorphous nature of the excipients (Figure 10a,b). In the case of physical mixture of the RSP/RM-β-CD inclusion complex with STR, the diffraction pattern reveals the disappearance of the high intensity crystalline reflection of the inclusion complex from 9.42 2*θ*; the other two characteristic peaks of the inclusion complex can be observed at 11.64 and 14.47 2*θ*, while the rest of the bands are overlapped over the broad bands of excipient. The PXRD pattern of the inclusion complex–CEL physical mixture exhibits an overlap of characteristic peaks of both the RSP/RM-β-CD inclusion complex observed at 9.45, 11.50, 14.29, 17.52, 19.90, 21.32 2*θ*, and the excipient, indicating a lack of interaction between the components.

The diffractogram of MgSTE shows characteristic crystalline reflections at 2*θ* of 5.35, 7.20, 8.90, 21.78, and 22.53, suggesting its crystalline state [18] (Figure 10c). The diffraction profile of physical mixtures of the inclusion complex with MgSTE represents a sum of characteristic peaks of KP that appear at 2*θ* of 9.42, 11.45, 14.27, 17.47, 19.84, and 21.30, and excipient peaks that appear at 5.41, 7.23, 9.02, 21.93, and 22.71 2*θ*, highlighting no interaction between the inclusion complex and MgSTE.

The crystalline profile of LCT is demonstrated by the high intensity crystalline reflections at 19.22, 19.69, 20.11, 20.77, and 21.09 2*θ* and other peaks with lower intensity at 10.61, 12.62, 16.53, 23.85, 24.75, 25.67, 31.85, and 33.32 2*θ*, which are present in its diffraction pattern (Figure 10d). The diffractogram of the inclusion complex–LCT mixture shows all the characteristic crystalline reflections of excipient (at 10.57, 12.58, 16.48, 19.17, 19.63, 20.06, 20.72, 21.03, 23.84, 24.71, 25.61, 31.78, and 33.24 2*θ*), but some corresponding to RSP/RM-β-CD KP are greatly attenuated (at 14.14 and 21.30 2*θ*) or shifted (9.36, 11.36, and 17.17 2*θ*), confirming the interaction in solid state between the inclusion complex and LCT that was previously demonstrated by thermal analysis and UATR-FTIR spectroscopy.

## 3. Materials and Methods

### 3.1. Materials

Risperidone (as Pharmaceutical Secondary Standard) was acquired from Sigma-Aldrich, (Steinheim, Germany) and randomly methylated β-cyclodextrin (DS~12) was purchased from Cyclolab R&L Ltd. (Budapest, Hungary). The pharmaceutical grade excipients, namely starch, microcrystalline cellulose, magnesium stearate, anhydrous lactose, and methylcellulose were obtained from Sigma-Aldrich (Steinheim, Germany). All other reagents and chemicals used were of analytical purity.

### 3.2. Phase Solubility Studies

Phase solubility studies were performed according to the method reported by Higuchi and Connors [38] in 0.1 M phosphate buffer of pH 7.4, at 25 °C. An excessive amount of RSP (10 mg) was added to 3 mL of solution containing RM-β-CD in concentration of 0–50 mM. The obtained suspensions were vigorously shaken at 25 °C for 5 days. After the equilibrium was reached, the samples were filtered using a 0.45 µm nylon disk filter and suitably diluted. The RSP concentration in filtered solutions was evaluated using UV spectroscopic measurements at 277 nm. Spectronic Unicam—UV 320 UV-Visible double beam spectrophotometer (Spectronic Unicam, Cambridge, UK) with 1 cm matched quartz cells was used for all UV spectrophotometric measurements.

The apparent stability constant (*K*_1:1_) was calculated from the phase solubility diagram, using the following equation:

(2) $$K_{1:1}=\frac{Slope}{S_{0}\;\left(1-Slope\right)}$$

where *S*_0_ is RSP solubility in phosphate buffer 0.1 M (pH 7.4) in the absence of RM-β-CD.

### 3.3. Molecular Docking Studies

Molecular docking studies were carried out to characterize the interaction between drug substance and CD. The RM-β-CD structure used in this work was generated from the curated coordinates of ligand 2QKH (X-ray diffraction, resolution 1.9 Å) downloaded from the Protein Data Bank database [59]. Methyl groups were manually added on free hydroxyl groups in order to obtain a degree of substitution equal with 12 (GaussView 5, Semichem Inc). Substituents were added on the β-cyclodextrin natural core, namely, 4-CH_3_ on the O_2_- position for the 2, 3, 4, and 6 glucopyranose units, 5 groups on the O_3_- for the 1, 2, 4, 5, and 7 glucose residues, and finally, 3-CH_3_ on 1, 5, and 7 glucopyranose units on the O_6_- position. All dihedral angles of the methoxy groups were homogenized, the resulting conformations being compatible with an unhindered CD cavity. CD was optimized in the same manner with RSP (DFT/B3LYP/6-311G). Three-dimensional coordinates of RSP was generated using the Gaussian program suite at DFT/B3LYP/6-311G optimization.

The molecular docking analysis was carried out using the Autodock 4.2.6 software together with the AutoDockTools [41]. The docking between RSP and RM-β-CD involves adding all the polar hydrogens, computing the Gasteiger charge; a grid box was created using Autogrid 4 with 50 × 50 × 50 Å in x, y and z directions with 0.375 Å spacing from CD center. All the calculations were performed in vacuum. For the docking process, we chose the Lamarckian genetic algorithm (Genetic Algorithm combined with a local search), with a population size of 150 and a number of 50 runs. In order to generate the molecular modeling figures, we exported all Autodock results in the PyMOL (The PyMOL Molecular Graphics System, Version 2.0 Schrödinger, LLC, New York, NY, USA) [43]. To validate the repeatability and reproducibility of the docking method, we performed redocking and then expressed the results as RMSD in Å using Discovery Studio software. All the calculations were performed in triplicate and expressed as an average.

### 3.4. Preparation of the Solid Inclusion Complex and Physical Mixtures with Excipients

The kneading method in a 1:1 molar ratio was employed to prepare the inclusion complex of RSP with RM-β-CD. For this purpose, 0.2851 g of RSP and 0.9149 g of CD were weighed, and the mixture was pulverized in an agate mortar and triturated with 1.2 g ethanol:HCl 0.1 M solution (1:1, m/m). Then, the thick slurry was kneaded for 45 min, and during the process, a few drops of solvent were added to maintain a suitable consistency. Thus, the product obtained was dried at ambient temperature and then in the oven at 40 °C for 24 h. The dried kneaded product was pulverized and passed through a 75-µm size sieve. In addition, a physical mixture of RSP with RM-β-CD in 1:1 molar ratio was obtained by mixing in the agate mortar and pestle for 10 min in a solvent-free manner.

The physical mixtures of RSP/RM-β-CD inclusion complex and each excipient were prepared by mixing in an agate mortar with pestle for approximately 5 min in the ratio of 1:1 (m/m).

### 3.5. Thermal Analysis

The pure RSP, RM-β-CD, and RSP/RM-β-CD physical mixture and kneaded product and also the mixtures of KP and selected excipients were analyzed using a Perkin-Elmer DIAMOND TG/DTA instrument. Samples with masses around 3–4 mg were weighed in aluminum crucibles and studied under air atmosphere at a flow rate of 100 mL/min, over the temperature range of 40–500 °C, with a heating rate of 10 °C/min.

### 3.6. Powder X-ray Diffractometry

PXRD studies were carried out using a Bruker D8 Advance powder X-ray diffractometer (Bruker AXS, Karlsruhe, Germany). The X-ray diffraction patterns were collected at ambient temperature, using CuKα radiation (40 kV, 40 mA) and a Ni filter, over the interval of 10–45° angular domain (2*θ*).

### 3.7. UATR-FTIR Spectroscopy

The UATR-FTIR spectroscopic analysis was performed using a Perkin Elmer SPECTRUM 100 device. The data were collected directly on solid samples in the spectral domain 4000–600 cm^−1^ on an UATR device. Spectra were built up after a number of 16 co-added acquisitions, with a spectral resolution of 4 cm^−1^.

### 3.8. Solubility Profile of RSP/RM-β-CD Kneaded Product

The saturation shake-flask method was employed in order to evaluate the RSP solubility upon complexation with RM-β-CD. To this end, an excessive amount of drug substance and RSP/RM-β-CD kneaded product were added in 5 mL of phosphate buffer 0.1 M (pH 7.4), so that saturated solutions were obtained. The samples were shaken for 24 h at room temperature, and then the solutions were separated from the insoluble drug substance by filtration using a 0.45 µm nylon disk filter. After appropriate dilution, the filtrate was subjected to UV-spectrophotometric analysis at 277 nm. The RSP quantification was realized using a calibration curve. A set of RSP solutions in phosphate buffer 0.1 M with concentration in the range of 10–70 μg/mL were prepared, and their absorbance was recorded at 277 nm. The standard curve was obtained by plotting the absorbance (*A*) vs. the concentration (*C*); the equation of the RSP calibration curve is: *A* = 0.02801·*C* + 0.00677, with *R* = 0.99981.

## 4. Conclusions

This study investigates the encapsulation of antipsychotic drug RSP by RM-β-CD and the compatibility of the inclusion complex with several excipients commonly used in pharmaceutical dosage forms. The RSP/RM-β-CD binary product was obtained using the kneading method and was evaluated by experimental and theoretical approaches. The experimental results provided by thermal methods, powder X-ray diffractometry, and UATR-FTIR spectroscopy demonstrate the inclusion complex formation between RSP and RM-β-CD in 1:1 molar ratio as the solubility studies indicated. As a result of inclusion complex formation, the RSP solubility was increased by 2.58-fold as compared with free RSP, highlighting the solubilizing effect of CD.

Since the corroboration of thermoanalytical data suggested that the inclusion complex is compatible with three of the selected excipients (namely STR, CEL, and MgSTE), but incompatible with LCT, two complementary investigational tools were used, namely UATR-FTIR spectroscopy and PXRD. These last two instrumental techniques allow the evaluation of compatibility/incompatibility between the components of a complex matrix in ambient conditions. It was shown that interactions between the RSP/RM-β-CD and LCT occur in solid state even under ambient conditions, and they are accentuated by the thermal stress. In the development of new generic forms containing RSP formulated as a supramolecular adduct with RM-β-CD, precautions should be taken in the selection of excipients, without using lactose in the final product.

## Figures and Tables

**Table 1:** UATR-FTIR characteristic bands for RSP/RM-β-CD and its mixtures with excipients.

Sample	Analysis of UATR-FTIR Spectral Regions (cm^−1^)		
	3600–2700	1700–1000	1000–650
RSP/RM-β-CD	3387; 2924	1642; 1535; 1450; 1415; 1364; 1272; 1194; 1154; 1083; 1037; 1006	959; 857; 758
RSP/RM-β-CD + STR	3386; 2919; 2847	1649; 1535; 1459; 1416; 1364; 1272; 1195; 1152; 1083;1035; 1006	962; 857; 758
RSP/RM-β-CD + CEL	3386; 2930	1649; 1535; 1458; 1416; 1364; 1272; 1194; 1155; 1083; 1034; 1009	956; 858; 758
RSP/RM-β-CD + MgSTE	2957; 2916; 2850	1639; 1572; 1541; 1465; 1418; 1365; 1272; 1192; 1154; 1083; 1038; 1007	961; 857; 722
RSP/RM-β-CD + LCT	3338; 2945; 2931; 2901	1642; 1535; 1420; 1369; 1254; 1195; 1157; 1142; 1118; 1083; 1068; 1031; 1018	989; 968; 877; 761

## Data Availability

Not applicable.

## Sample Availability

Samples of the compounds are available in limited quantity from the authors.
